# Effect of prostate-specific membrane antigen positron emission tomography on the decision-making of radiation oncologists

**DOI:** 10.1186/s13014-015-0548-8

**Published:** 2015-11-18

**Authors:** Thomas P. Shakespeare

**Affiliations:** Department of Radiation Oncology, North Coast Cancer Institute, 345 Pacific Highway, Coffs Harbour, NSW 2450 Australia; University of New South Wales Rural Clinical School Faculty of Medicine, 345 Pacific Highway, Coffs Harbour, NSW 2450 Australia

**Keywords:** Prostate cancer, Radiotherapy, Prostate-specific membrane antigen, Positron emission tomography, Decision-making, Staging, Radiation oncologist

## Abstract

**Background:**

Positron emission tomography (PET) imaging is routinely used in many cancer types, although is not yet a standard modality for prostate carcinoma. Prostate-specific membrane antigen (PSMA) PET is a promising new modality for staging prostate cancer, with recent studies showing potential advantages over traditional computed tomography (CT), magnetic resonance imaging (MRI) and nuclear medicine bone scan imaging. However, the impact of PSMA PET on the decision-making of radiation oncologists and outcomes after radiotherapy is yet to be determined. Our aim was to determine the impact of PSMA PET on a radiation oncologist’s clinical practice.

**Findings:**

Patients in a radiation oncology clinic who underwent PSMA PET were prospectively recorded in an electronic oncology record. Patient demographics, outcomes of imaging, and impact on decision-making were evaluated.

Fifty-four patients underwent PSMA PET between January and May 2015. The major reasons for undergoing PET included staging before definitive (14.8 %) or post-prostatectomy (33.3 %) radiotherapy, and investigation of PSA failures following definitive (16.7 %) or post-prostatectomy (33.3 %) radiotherapy. In 46.3 % of patients PSMA was positive after negative traditional imaging, in 9.3 % PSMA was positive after equivocal imaging, and in 13.0 % PSMA was negative after equivocal imaging. PSMA PET changed radiotherapy management in 46.3 % of cases, and hormone therapy in 33.3 % of patients, with an overall change in decision-making in 53.7 % of patients.

**Conclusions:**

PSMA PET has the potential to significantly alter the decision-making of radiation oncologists, and may become a valuable imaging tool in the future.

## Findings

### Introduction

Positron emission tomography (PET) imaging is a common staging tool in a variety of malignancies, with early investigations demonstrating the potential utility of prostate-specific membrane antigen (PSMA) for the detection of prostate cancer [1, 2]. PSMA is a very specific prostate epithelial cell membrane antigen which is significantly over-expressed in prostate cancer cells compared to other PSMA-expressing tissues such as kidney, proximal small intestine, and salivary glands [3]. Unlike PSA, PSMA is membrane-bound and not secreted [4]. These characteristics make it an ideal extracellular target for imaging modalities [5].

Early studies have shown that PSMA PET has sensitivity, specificity, negative predictive values (NPV) and positive predictive values (PPV) of up to 76.6, 100, 91.4 and 100 % respectively [1]. A critical review of the PET literature [2] compared PSMA with four other tracers (^11^C- or ^18^F-choline, ^11^C-acetate, anti-1-amino-3-^18^F-fluorocyclobutane-1-carboxylic acid, and ^18^F-fluorodeoxyglucose) in the detection of prostate cancer. This review demonstrated a potential advantage for the use of PSMA over other PET tracers for prostate cancer, with PSMA having a greater likelihood of detecting lymph node and bone lesions. However the authors noted that at the time of the review in 2014, there was fairly limited data on the use of PSMA.

Since the publication of Yu’s review, several studies have demonstrated the potential benefits of PSMA PET in identifying prostate cancer recurrences after surgery [1, 6] and radiotherapy [1]. In these studies PSMA PET was shown to convert PSA-only failures to metastatic failures through the early detection of recurrences. The ability of PSMA-PET to change the known outcome of treated prostate cancer might conceivably have an impact on the decision-making of clinicians, and potentially change management approaches. There is one published report of 22 patients evaluating the effect of PSMA PET on the clinical decision-making of urologists [5], showing it “significantly impacted” upon how patients were managed. However to date there has been no evaluation of the potential effects of PSMA PET on decision-making from a radiation oncology perspective.

The aim of this study was to evaluate how PSMA PET changed the decision-making process and management outcomes within a radiation oncology clinic.

## Methods

Between January and May 2015, a total of 54 patients attending a radiation oncology clinic were referred for PSMA-PET imaging. Patients were only considered for PET if conventional staging including CT, bone scan and/or MRI was not definitive, if there was a high clinical suspicion despite negative or equivocal imaging, or if patients were being considered for radiotherapy to oligometastatic disease (defined as 1–3 nodal or distant metastases). Intended management of patients was based on standard departmental protocol. Patients with PSA-only failures under 10 were planned for observation. Those with PSA failures above 10, or PSA <10 with demonstrated systemic metastases on imaging were planned for treatment with androgen deprivation therapy (ADT). Patients with oligometastatic disease were planned to have high dose radiotherapy +/− systemic therapy.

All patients undergoing PSMA PET had tomographic images obtained from the skull vertex to knees following the intravenous injection of 159 MBq Ga-68 ligand (Ga-68 PSMA). A low dose CT scan was performed using tidal respiration for attenuation correction and lesion localisation.

Patients sent for PSMA PET were prospectively tagged within an electronic oncology record system. Patient files were retrospectively evaluated for PET outcome and management received. This retrospective review completed institutional ethics processes and was assigned the reference number QA101. Uni- and multivariate analyses were performed using binary logistic regression for the variables patient age, initial PSA, Gleason score and stage, and pre-PET PSA.

## Results

Patient demographics and reasons for PSMA PET are shown in Table 1. Of note 26 (48.1 %) of patients were being considered for curative doses of radiotherapy (either in the definitive or post-prostatectomy setting), and 27 (50.0 %) were to investigate PSA failures after definitive or post-prostatectomy radiotherapy, with a view to considering high dose radiotherapy if oligometastatic disease was found.

**Table 1:** Patient demographics

Age (years)	Median	69
	Range	52–83
Gleason†	6–7	33 (61.1 %)
	8–9	20 (37.0 %)
Initial PSA	Median	9.15
	Range	1.3–36.0
Pre-PSMA PSA	Median	1.1
	Range	0.017–20.4
Reason for PET n (%)	Pre-radical IMRT staging	8 (14.8 %)
	Pre-PPRT staging	18 (33.3 %)
	PSA failure after IMRT	9 (16.7 %)
	PSA failure after PPRT	18 (33.3 %)
	Response to systemic therapy	1 (1.9 %)

†1 missing

IMRT = intensity-modulated radiotherapy

PPRT = post-prostatectomy radiotherapy

The effect of PSMA PET on disease status outcomes and decision-making are shown in Table 2. In 46.3 % of patients PSMA was positive when conventional imaging was negative. In 13.0 % of cases, equivocal conventional imaging was negative on PSMA, and in 9.3 %, equivocal conventional imaging was positive on PSMA.

**Table 2:** Effect of PSMA PET on disease status outcomes and radiation oncologist decision-making

PSMA vs Conventional scans	PSMA-/CS-	17 (31.5 %)
	PSMA+/CS-	25 (46.3 %)
	PSMA-/CS+	7 (13.0 %)
	PSMA+/CS+	5 (9.3 %)
Change in RT management	No	29 (53.7 %)
	Yes	25 (46.3 %)
Change in ADT management	No	36 (66.7 %)
	Yes	18 (33.3 %)
Any change in management	No	25 (46.3 %)
	Yes	29 (53.7 %)

CS = conventional scans (CT, bone scan, MRI)

- = Negative

+ = Equivocal

RT = radiotherapy

ADT = androgen deprivation therapy

In all, 53.7 % of patients had a change in management due to the PSMA PET (Table 2), with 46.3 % having a change in radiotherapeutic management, and 33.3 % a change in ADT management. Treatment plans pre- and post-PET are shown in Table 3. Of particular note, prior to PET 50.0 % were planned for observation and this reduced to 18.5 % after PET. In addition 9.3 % were planned for treatment to oligometastases pre-PET and this increased to 37.0 % post PET. Several of the patients who were planned for oligometastatic treatment pre-PET had the number and/or location of sites changed as a result of PSMA PET.

**Table 3:** Treatment plan before and after PSMA PET

Treatment plan	Pre-PET (%)	Post-PET (%)
Observe	50.0	18.5
Radical radiotherapy (to prostate or prostate bed)	31.5	27.8
Radical radiotherapy including nodes	1.9	0
Palliative radiotherapy to oligometastases	9.3	37.0
Systemic therapy alone	7.4	16.7

On uni- and multivariate analysis, Gleason score was the only significant predictor. The odds of PET changing decision-making for Gleason 8–10 disease was 7.6 times that of Gleason 6–7 disease (*p* = 0.01).

## Discussion

This study has shown that when PSMA PET imaging is utilised for patients seen in a radiation oncology clinic, there can be a substantial effect on radiation oncologist decision-making. The heterogeneous group of patients in our cohort are consistent with what would be expected to be encountered in a radiation oncology clinic, and strengthens the applicability of our findings to daily practice. In our study there was a particular benefit for patients with PSA failures post definitive or salvage radiotherapy, and to clarify equivocal findings of conventional imaging. The only other study investigating the effect of PSMA PET on decision-making was by Demirkol [5]. This study of 22 patients being managed by urologists also found a significant impact on the management of patients.

In our study, potentially curable patients were found to be incurable, and potentially incurable patients were found to be curable. Sites for radiotherapy targeting differed, doses were occasionally altered, and some patients in the post-prostatectomy radiotherapy setting were so reassured by a negative PSMA that they opted not to have any therapy at all (despite recommendations to the contrary). Equivocal findings on conventional imaging were often found to be negative on PSMA, confirming a treatment plan even though not altering it. This highlights the fact that although PSMA PET appears promising, further research is required to confirm its accuracy and limitations.

One interesting area of potential benefit is the use of PSMA PET in patients being considered for high dose radiotherapy for oligometastatic disease. This is an area of significant interest for radiation oncologists [7], and PSMA PET appears to have the potential to identify early oligometastatic disease as well help aid targeting of radiotherapy, although whether this improves patient outcomes is unknown. This is an area ripe for further investigation.

Another area where PSMA PET may be useful is in detecting systemic disease to allow earlier administration of systemic therapy in the clinical trial setting. Effectively PSMA PET has the potential to change outcomes (changing disease status from PSA-only failure with conventional imaging to metastatic failure). This change may have an adverse effect on published metastasis-free survival outcomes after radical treatments. However with earlier oligometastatic and systemic therapy initiation, this could theoretically improve prostate-cancer specific and overall survival. It is clear that further research is required not only to evaluate the diagnostic efficacy of PSMA PET, but to determine whether earlier detection and treatment of disease (regardless of the modality of diagnostic imaging used) translates into survival or quality-of-life benefits.

In conclusion, in our cohort PSMA PET had a significant impact on radiation oncologist decision-making, and impacted on patient management outcomes.
